# A functional role for the cancer disparity-linked genes, CRYβB2 and CRYβB2P1, in the promotion of breast cancer

**DOI:** 10.1186/s13058-019-1191-3

**Published:** 2019-09-11

**Authors:** Maya A. Barrow, Megan E. Martin, Alisha Coffey, Portia L. Andrews, Gieira S. Jones, Denise K. Reaves, Joel S. Parker, Melissa A. Troester, Jodie M. Fleming

**Affiliations:** 1 Department of Biological and Biomedical Sciences, North Carolina Central University, 1801 Fayetteville Street, Mary Townes Science Complex, Durham, NC 27707 USA; 2 Lineberger Comprehensive Cancer Center, University of North Carolina at Chapel Hill, Chapel Hill, NC USA; 3 Department of Genetics, University of North Carolina at Chapel Hill, Chapel Hill, NC USA; 4 Department of Epidemiology, University of North Carolina at Chapel Hill, Chapel Hill, NC USA

**Keywords:** Crystallin beta B2, Crystallin beta B2 pseudogene 1, Breast cancer, Cancer health disparities

## Abstract

**Background:**

In the USA, the breast cancer mortality rate is 41% higher for African-American women than non-Hispanic White women. While numerous gene expression studies have classified biological features that vary by race and may contribute to poorer outcomes, few studies have experimentally tested these associations. *CRYβB2* gene expression has drawn particular interest because of its association with overall survival and African-American ethnicity in multiple cancers. Several reports indicate that overexpression of the *CRYβB2* pseudogene, *CRYβB2P1*, and not *CRYβB2* is linked with race and poor outcome. It remains unclear whether either or both genes are linked to breast cancer outcomes. This study investigates *CRYβB2* and *CRYβB2P1* expression in human breast cancers and breast cancer cell line models, with the goal of elucidating the mechanistic contribution of CRYβB2 and *CRYβB2P1* to racial disparities.

**Methods:**

Custom scripts for *CRYβB2* or *CRYβB2P1* were generated and used to identify reads that uniquely aligned to either gene. Gene expression according to race and tumor subtype were assessed using all available TCGA breast cancer RNA sequencing alignment samples (*n =* 1221). In addition, triple-negative breast cancer models engineered to have each gene overexpressed or knocked out were developed and evaluated by in vitro, biochemical, and in vivo assays to identify biological functions.

**Results:**

We provide evidence that *CRYβB2P1* is expressed at higher levels in breast tumors compared to *CRYβB2*, but only *CRYβB2P1* is significantly increased in African-American tumors relative to White American tumors. We show that independent of CRYβB2, *CRYβB2P1* enhances tumorigenesis in vivo via promoting cell proliferation. Our data also reveal that *CRYβB2P1* may function as a non-coding RNA to regulate CRYβB2 expression. A key observation is that the combined overexpression of both genes was found to suppress cell growth. CRYβB2 overexpression in triple-negative breast cancers increases invasive cellular behaviors, tumor growth, IL6 production, immune cell chemoattraction, and the expression of metastasis-associated genes. These data underscore that both CRYβB2 and *CRYβB2P1* promote tumor growth, but their mechanisms for tumor promotion are likely distinct.

**Conclusions:**

Our findings provide novel data emphasizing the need to distinguish and study the biological effects of both CRYβB2 and CRYβB2P1 as both genes independently promote tumor progression*.* Our data demonstrate novel molecular mechanisms of two understudied, disparity-linked molecules.

**Electronic supplementary material:**

The online version of this article (10.1186/s13058-019-1191-3) contains supplementary material, which is available to authorized users.

## Background

While breast cancer is the most common cancer among women, a survival gap exists among African-American and Caucasian/White women [1]. This disparity has persisted over the last decade, despite notable improvements in survival for both races. Historically, White women of all ages exhibited higher incidence; however, recent data suggest that overall incidence rates have converged [1, 2]. Socioeconomic and other factors, including timely access to care, the quality of care, diet and exercise, as well as environmental, and biological factors have been cited as potential explanations of the survival disparity [3, 4]. For example, a higher prevalence of Basal-like breast cancers, a subtype associated with poorer prognosis, is often touted as a prime contributor to the higher mortality rates in young African-American women [5, 6]. However, recent studies stress that African-American women also have higher mortality within luminal A breast cancers and a higher risk of recurrence scores among estrogen receptor-positive and HER2-negative breast cancers [7–9]. To this point, there have been numerous gene expression studies that have classified biological features that vary by race. These inherent biological differences within the tumors may attribute to poorer outcomes witnessed among African-Americans, though few of these observational studies have experimentally tested these associations [4, 7, 10–13]. Accordingly, these reports underscore the need for more basic mechanistic studies to test the contribution of these biological factors to disease progression and patient outcome.

Crystallin β B2 (CRYβB2) has recently drawn particular interest because of its genetic association with overall survival and African-American ethnicity in multiple cancers, including breast, colorectal, renal cell carcinoma, glioblastoma, and prostate tumors [7, 11, 12, 14–17]. In a small prediction analysis study, 91% of all African-American/Black patients (*n* = 33) and 94% of all White patients (*n* = 36) were correctly classified according to race using *CRYβB2* as one of the two-gene signatures in prostate tumors, *PSPHL* being the other gene classifier [12]. Similar prediction analyses have been performed using colorectal and breast tumors [7, 11, 14, 15, 18]. Additional studies have also revealed *CRYβB2* to be differentially expressed in non-malignant African-American breast tissue [7, 14]. Thus, this gene has successfully been used as a classifier to distinguish between racial groups. Further, higher *CRYβB2* expression has been correlated to poorer outcome in cancer, regardless of race [7, 11, 12, 14–16]. Even with these findings, no study has demonstrated a functional role for *CRYβB2* in cancers.

The CRYβB2 protein is an abundant ocular lens protein, and mutations have been associated with congenital cataracts and macular degeneration [19, 20]. Mouse model studies have also demonstrated Crybb2^−/−^ mice have reduced fertility compared with wild-type mice via reduced expression of cell cycle and survival genes [21, 22]. Critical to this study, previous reports have indicated that the *CRYβB2* pseudogene, *CRYβB2P1*, and not the parental gene was linked with the observed poor outcome in African-American breast cancers and congenital cataracts in particular ethnicities/populations [23–27]. Pseudogenes are copies of protein-coding genes that no longer produce the same functional product as their parental gene, but still share a high-sequence similarity, and can thus regulate or mediate the function of their parental genes through mechanisms such as the generation of non-coding RNAs (ncRNA). An emergent body of literature clearly shows that pseudogenes perform vital roles in regulating normal tissue growth and the development of some diseases, especially cancers [28]. A critical evaluation of published reports identified that the majority of gene expression microarrays examined indiscriminately detect both *CRYβB2* and *CRYβB2P1*, due to their high sequence similarity. Therefore, the potential exists that *CRYβB2P1* expression has confounded prior results. It remains unclear whether either gene, or both genes, is linked to breast cancer outcomes. This study investigates racial expression differences and regulatory relationship between *CRYβB2* and *CRYβB2P1*.

## Methods

### Dataset and data processing: re-quantification of *CRYβB2* and *CRYβB2P1*

All available Cancer Genome Atlas (TCGA) breast cancer RNA sequencing alignment files (*n* = 1221) were retrieved from GDC Data Portal (https://portal.gdc.cancer.gov). Sequence data aligned to chromosome 22: 25,216,000–25,525,000, which span the *CRYβB2* and *CRYβB2P1* genes were extracted for further study. Custom scripts were used to search through the alignment files and identify reads that aligned to either *CRYβB2* or *CRYβB2P1*, or both genes. The genomic coordinates for each position of each read were marked as either uniquely-mapped to either gene, or as part of a multi-mapped region. Unique and multi-mapped positions from each alignment file were combined, and those that were consistently unique across all samples were merged to form composite regions of unambiguous alignments. The algorithm was tested and validated by adding synthetic reads that mapped to unique regions for each gene and in the multi-mapped regions to several alignment files. Only after validating each stage of the process was the algorithm applied, and the results were used for downstream analyses.

PySam 0.15.0, a python wrapper for Samtools, was used to quantify the reads that mapped to any of the composite unique regions in either *CRYβB2* or *CRYβB2P1*. Since the RNA sequencing reads were 50 nucleotides long, only unique regions that were greater than 50 nucleotides in length were used to quantify the reads. Reads that were flagged in the alignment files as unmapped, having failed quality checks, secondary reads, or PCR duplicates were excluded. Re-quantified counts for each gene were combined with un-normalized counts for all genes in TCGA breast cancer RNA sequencing, and upper quartile normalized. Normalized counts were log2(*x* + 1) transformed and used for analysis unless specified otherwise.

### Cell lines, generation of expression models, cell proliferation, and chemoattraction assays

The SUM159 cell line was obtained from Asterand (Detroit, MI) and all remaining cell lines from American Type Culture Collection (Manassas, VA, Additional file 1: Table S1) with the provided authentication documents, cultured as directed by manufacturers. Cells were authenticated every 6 months via short tandem repeat (STR) profiling. Overexpression lentiviral particles and CRIPSR expression vectors were obtained from Genecopoeia (Rockville, MD) and cell transduced or transfected according to the manufacturer’s instructions. Transfection was conducted using the Fugene HD reagent (Promega) as directed. Clonal cell lines were established via flow cytometry and antibiotic selection. Proliferation assays were conducted as previously described. Briefly, cells were plated in triplicate for each time point, and at the predetermined concentration for each cell line [29]. Cell counts were taken every 24 h for a total of 96 h using a TC20™ automated cell counter (Bio-Rad, Hercules, CA). Cell chemoattraction assays: Costar Transwell permeable support 3.0-μm polycarbonate membranes were used according to the manufacturer’s protocol. The indicated cell model was plated and grown to 80% confluence on 24-well dishes, washed, then placed in serum-free media for 24 h to condition the media. U937 (ATCC) cells were washed, resuspended in serum-free medium, and plated in the top chamber of Transwell inserts (1 × 10^5^ cells per insert; each model plated in duplicate). Cells migrated through the membrane for 4 h towards the indicated cell model in 24-h conditioned media. After this time, non-migratory cells were wiped from the top surface of the membrane; migratory cells were then fixed in methanol and stained with 1.0% crystal violet. Cell numbers were determined from microphotographs taken over five (non-overlapping) areas of the membrane.

### Western immunoblotting and immunocytofluorescence

Cells were lysed in mPer Lysis Buffer (Thermo Scientific, Rockford, IL) supplemented with protease and phosphatase inhibitors (Halt™ Thermo Scientific), then subjected to western analyses as previously described [29–31]. Antibodies: CRYBB2, Novus Biologicals (Centennial, CO; catalogue numbers 001415-M02 and NBP2-13876) and STAT3, Phospho-Stat3^705^, Actin, Cell Signaling Technology (Danvers, MA; catalogue numbers 9131, 9145, and 4968, respectively). Immunofluorescence was performed with appropriate controls as previously described [32].

### Quantitative real-time PCR, subcellular RNA fractionation, and detection of metastatic cells

Total RNA was isolated using the RNeasy kit (Qiagen; Hilden, Germany). Subcellular RNA fractionation was performed using the Active Motif kit #25501 (Carlsbad, CA) following the manufacturer’s instructions with the modification of lysis shortened to 2 min. RNA was reverse transcribed using the High Capacity cDNA RT Kit (Life Technologies), and qPCR analysis conducted using the Absolute Blue qPCR Mix, Low Rox (ThermoFisher) with the Applied Biosystems QuantStudio™ 6 Flex Real-Time PCR system each according to the manufacturer’s instructions. Relative fold changes in gene expression were determined via the ^ΔΔ^CT method and/or the standard curve analysis when indicated. Primer sequences are listed in Additional file 2: Table S2.

### Xenograft generation

Animal experiments were conducted in accordance with accepted standards of humane animal care and approved by the Animal Care and Use Committee at North Carolina Central University. Xenografts were generated as we previously described [33]. Briefly, 5-week-old female Hsd:Athymic Nude-*Foxn1*^*nu*^ mice (Envigo, Dublin, VA) were injected orthotopically into the right abdominal mammary gland with 5 × 10^5^ of the indicated cell model suspended in 30.0 μl of 50/50 PBS:Matrigel. Weekly tumor growth was measured via calipers and tumors excised when volume neared 400 mm^3^. Whole tumors were homogenized and RNA extracted using the Qiagen RNeasy kit as instructed. Three independent experiments were performed to ensure repeatability. Mice that did not form tumors were euthanized 5 months post-injection. Detection of metastatic xenograft cells in liver and lung was conducted as previously described [33].

### Three-dimensional morphogenesis assays and live cell imaging

3D cultures were performed as previously described [34–36]. Briefly, equal numbers of proliferating cells were plated on laminin-rich basement membrane gels (growth factor-reduced Matrigel®, Sigma) in culture medium containing 2.0% basement membrane to support 3D growth; 3D growth medium was replaced every 3 days. Cells were imaged on the 10th day of culture, and cell morphology, size/area, organization, and growth were evaluated using a Nikon DiaPhot microscope with digital camera and NIS-Elements 4.11.00 (Nikon Instruments Inc., NY). Live cell imaging was conducted using an IncuCyte® S3 Live Cell Analysis System (Sartorius) using phase contract, × 10 magnification, one image captured every 4 h for 3 days. Cells were extracted from gels for RNA isolation using the Corning Cell Recovery Solution as instructed.

### IL6 signaling inhibition and IL6 ELISAs

IL6 signaling was inhibited using a 24-h dose of 75.0 ng/ml in vitro and 100.0 μg/kg in vivo intratumoral injection of a recombinant human IL6 receptor blocking antibody or isotype control (R&D Systems, catalogue MAB227 and MAB002) [37]. Inhibition of IL6 signaling was confirmed via STAT3 phosphorylation. ELISA: Proliferating cells were washed and cultured in serum-free media for 24 h. Conditioned medium was collected, centrifuged to remove residual cells, and concentrated via Millipore Amicon™ Ultra-15 Centrifugal Filter Units. Medium was normalized for total protein content and quantitated for total IL6 using the RayBio® Human IL6 ELISA Kit as instructed.

### Additional statistical analysis

For all assays, a minimum of three independent experiments were performed using a minimum of duplicate samples in each experiment. For correlation determination, the Spearman correlation was used. Significance was determined via one-way analysis of variance (ANOVA) with Tukey Honest Significant Difference (HDS) or Bonferroni post hoc analyses. *T* tests were also performed using GraphPad Prism Software 6.0 unless otherwise noted. Data was considered significant at *P* < 0.05.

## Results

### Evaluating *CRYβB2* and *CRYβB2P1* gene expression differences in TCGA breast cancer samples by subtype and race

**Fig. 1:**
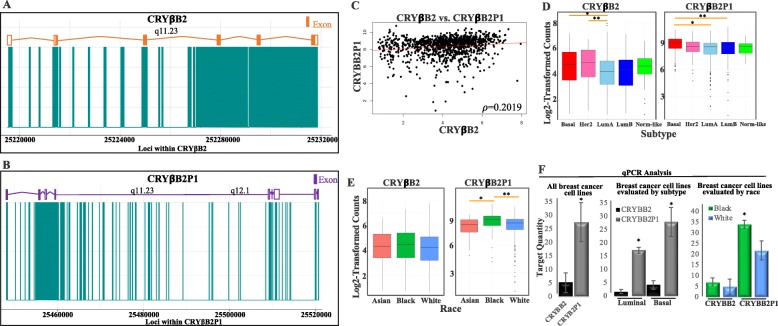
Correlation between *CRYβB2* and *CRYβB2P1*, and distribution of each gene among subtype and among race. **a**, **b** Unique regions and those that are common between *CRYβB2* and *CRYβB2P1* (referred to as multi-mapped) are depicted. Multi-mapped regions are shaded in blue, whereas unique regions are not shaded. The number and location of exons are indicated in orange for CRYβB2 protein-coding regions, and purple for all processed transcripts of *CRYβB2P1*. **c**–**e** Read counts were generated from the unique regions of the respected genes within all TCGA breast cancer samples, then normalized using upper quartile normalization. **c** Correlation between *CRYβB2* and *CRYβB2P1*. **d** Boxplots showing the distribution of each gene by subtype. *CRYβB2* *adjusted *p* = 0.0027, **adjusted *p* = 0.0099; CRY*β*B2P1 *adjusted *p* = 1.2E−07, **adjusted *p* = 1.8E−04. **e** Boxplots showing the distribution of *CRYβB2P1* by race *adjusted *p* = 5.8E−04, **adjusted *p* = 1.9E−06. **f** qPCR standard curve analysis of 24 breast cancer cell lines. Data represent mean ± SEM *p < 0.01 for *CRYβB2P1* in Black cell lines over *CRYβB2P1* in White and *CRYβB2* in all cell lines

To determine whether there is an independent contribution of *CRYBB2* and *CRYBB2P1* to the promotion of breast cancer and breast cancer disparities, custom scripts were used to search through all available TCGA breast cancer RNA sequencing alignment files (*n* = 1221) to identify reads that aligned to either *CRYβB2* or *CRYβB2P1*, or both genes. The genomic coordinates for each position of each read were marked either as uniquely mapped to either gene or as part of a multi-mapped region (visualized in Fig. 1a, b). Unique and multi-mapped positions from each alignment file were combined, and those that were consistently unique across all samples were merged to form composite regions of alignments.

Patient demographics and tumor subtypes (Table 1) were assessed for associations with the abundance of unambiguous alignments. Samples were first stratified according to race with Caucasian/White women constituting 77.6%, African-American/Black 16.9%, and Asian 5.5% of the total sample set. The luminal A subtype was the most numerous in White (52.6%) and Asian (33.9%) tumor samples, while Basal tumors were the most numerous subtypes for Black samples (33.9%) compared to all other subtypes evaluated. Assessment of correlation between the unambiguous expression estimates of *CRYβB2* and *CRYβB2P1* resulted in no significant correlation observed between *CRYβB2* and *CRYβB2P1* among all samples (Fig. 1c, *p* = 0.2019; Spearman Correlation). Overall, there were consistently higher counts for *CRYβB2P1* compared to *CRYβB2* in all breast tumor subtypes and ethnicities evaluated (Fig. 1d, e). *CRYβB2* expression was significantly higher in the basal and Her2 tumors compared to luminal A tumors (adj *p* = 0.003 and 0.009, respectively; one-way ANOVA with Tukey HSD test), while *CRYβB2P1* expression was significantly higher in the basal tumors compared to luminal A and B tumors (adj *p* = 1.2E−07 and 1.80E−04, respectively; one-way ANOVA with Tukey HSD test). Divergent from previous reports, the levels of *CRYβB2* were not significantly different between Asian, Black, and White tumors. However, *CRYβB2P1* had significantly higher expression in Black tumors compared to Asian and White (Fig. 1e, adj *p* = 5.8E−04 and adj *p* = 1.9E−06, respectively; one-way ANOVA with Tukey HSD test). These data correspond with a subset of studies that suggest *CRYβB2P1*, and not *CRYβB2*, is the gene associated with health disparities and poor outcome in breast cancers [23, 24]. When evaluating the data by first conditioning based on either subtype or race, the same patterns of significance persisted for each gene with only one exception; *CRYβB2* expression was no longer significantly higher in Her2 compared to luminal A tumors (Additional file 3: Figure S1A&B). The participant demographic factors of age/menopause status were not significant when observing gene distribution among race or subtype for *CRYβB2* (*not shown*). However, *CRYβB2P1* was significantly differentially expressed by race as well as by subtype after conditioning for race when evaluating age/menopause status (Additional file 3: Figure S1C-E). Most notably, *CRYβB2P1*was significantly higher in postmenopausal Black tumor samples compared to White postmenopausal and perimenopausal, and Asians under 40 years of age (adj. *p* = 0.027, 0.005, and 0.033, respectively; one-way ANOVA with Tukey HSD test). To test if similar patterns of expression were observed in breast cancer cell lines, a panel of 24 luminal and basal breast cancer cell lines were evaluated via qPCR using primers specific to either *CRYβB2* or *CRYβB2P1* (cell lines and race are listed in Additional file 1: Table S1)*.* Results corresponded with the patterns observed in tumor samples, with increased transcript abundance of *CRYβB2P1*in basal and Black cell models compared to *CRYβB2* (*p <* 0.01, Fig. 1f).

**Table 1:** Demographic and tumor characteristics

Subtype	Asian (*n = 62*)	Black (*n = 189*)	White (*n = 867*)	Total (*N* = 1118)
Basal	7 (11.3%)	64 (33.9%)	113 (13.0%)	184 (16.5%)
Her2	16 (25.8%)	16 (8.5%)	39 (4.5%)	71 (6.4%)
Luminal A	21 (33.9%)	62 (32.8%)	456 (52.6%)	539 (48.2%)
Luminal B	16 (25.8%)	31 (16.4%)	137 (15.8%)	184 (16.5%)
Normal like	2 (3.2%)	16 (8.5%)	122 (14.1%)	140 (12.5%)
Race	Young (*n = 76*)	Pre (*n = 111*)	Peri (*n = 274*)	Post (*n = 551*)
Asian	8 (10.5%)	6 (5.4%)	23 (8.4%)	24 (4.4%)
Black	20 (26.3%)	18 (16.2%)	59 (21.5%)	86 (15.6%)
White	47 (61.8%)	77 (69.4%)	192 (70.1%)	441 (80.0%)

Young, < 40 years; *Pre* pre-menopausal, 40–46; *Peri* peri-menopausal, 46–55; *Post* post-menopausal, > 55 years

### Loss of *CRYβB2P1* expression increases CRYβB2 levels

**Fig. 2:**
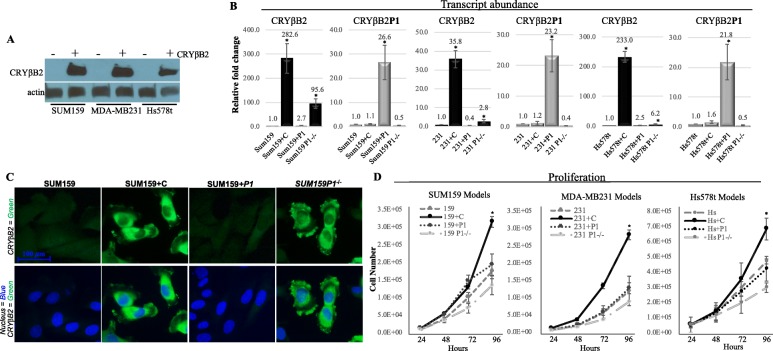
Expression patterns and proliferation rates of triple-negative breast cancer cells with altered CRYβB2 and *CRYβB2P1*. **a**, **b** Immunoblot and qRT-PCR analysis of the indicated cell lines with altered CRYβB2 and *CRYβB2P1*. Overexpression and knockout clonal populations were generated and selected via lentiviral transduction and CRISPR/Cas9-mediated transfection, flow cytometry, and antibiotic selection. **c** Immunofluorescence demonstrating the localization and expression of CRYβB2 in the SUM159 cell models was performed using an anti-CRYβB2 primary antibody and an anti-rabbit Alexa Fluor-488(green)-labeled secondary antibody. DNA; DAPI (blue), imaged at × 40. **d** Cells were plated at 25,000 cells per well in triplicate and counted every 24 h for 96 h. All data represent mean ± SE of a minimum of three independent experiments. *p < 0.01. 231, MDA-MD231; +C, CRYBB2 overexpression; +P1, *CRYβB2P1* overexpression; P-/-, *CRYβB2P1* knockout

To study the independent effects of each gene, overexpression of *CRYβB2* and *CRYβB2P1* and CRISPR knockout of *CRYβB2P1* cell models from three triple-negative breast cancer (TNBC) cell lines were established (Fig. 2). Basal levels of CRYβB2 protein in the parental cell models were below detectable levels via immunoblotting from cell cultures growing in 2D but were readily detectable in the overexpression models (Fig. 2a). The most striking observation in all cell models was that the knockout of *CRYβB2P1* resulted in a significant increase of transcript abundance as well as protein expression of CRYβB2 (*p <* 0.01, *t* test; Fig. 2b, c)*.* CRYβB2 was primarily localized to the cytoplasm and pseudopodial structures in both the CRYβB2 overexpression and *CRYβB2P1* knockout models. Proliferation assays demonstrated that overexpression of CRYβB2 significantly enhanced cell proliferation compared to the control parental cells as well as *CRYβB2P1* overexpression or knockout models (*p <* 0.01, one-way ANOVA with Bonferroni test; Fig. 2d). Thus, while *CRYβB2P1* knockout increased CRYβB2 expression, it was not sufficient to produce the enhanced proliferation observed in the CRYβB2 overexpressing models.

**Fig. 3:**
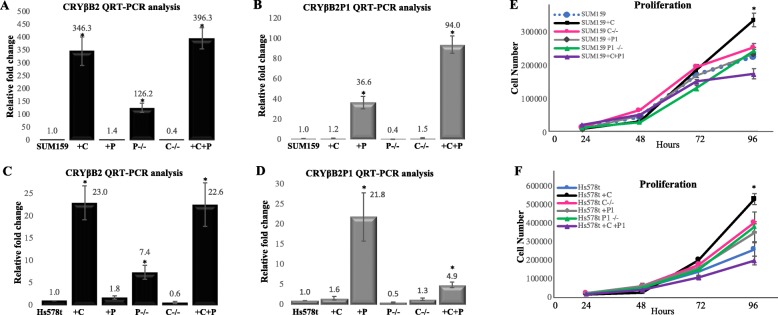
Expression and proliferation of SUM159 and Hs578t models including double overexpression of CRYβB2/*CRYβB2P1* and *CRYβB2* knockout. **a**–**d** qRT-PCR analysis of the indicated cell lines with altered CRYβB2 and CRYβB2P1. Overexpression and knockout clonal populations were generated and selected via lentiviral transduction and CRISPR/Cas9-mediated transfection, flow cytometry, and antibiotic selection. The indicated gene transcript abundance was measured via qRT-PCR. **e**, **f** Proliferation assays: cells were plated at 25,000 cells per well in triplicate and counted every 24 h for 96 h. All data are mean ± SE of a minimum of three independent experiments. *p < 0.05. +C, CRYβB2 overexpression; +P1, *CRYβB2P1* overexpression; P-/-, *CRYβB2P1* knockout; C-/-, *CRYβB2* knockout; +C+P, CRYβB2 and *CRYβB2P1* dual overexpression

As expression of *CRYβB2P1* repressed expression of CRYβB2, CRISPR knockout models of *CRYβB2* and dual overexpression of models of *CRYβB2* and *CRYβB2P1* were generated to obtain a comprehensive analysis of the gene interactions (Fig. 3). CRYβB2 overexpression and knockout did not alter *CRYβB2P1* transcript abundance. These data suggest that while *CRYβB2P1* may function as a non-coding RNA to regulate transcription, CRYβB2 does not have a significant reciprocal effect on *CRYβB2P1* expression. Cellular fractionation also confirmed that loss of either gene did not alter transcript localization between the nucleus and cytosolic fractions (Additional file 3: Figure S2). Proliferation assay results show that the dual overexpression of *CRYβB2* and *CRYβB2P1* resulted in the lowest proliferation rates for the Hs578t and SUM159 models tested, and CRY*β*B2 overexpression remained the only genetic alteration capable of significantly altering proliferation in 2D cultures (*p* < 0.05, one-way ANOVA with Bonferroni test; Fig. 3e, f).

### CRYβB2 and *CRYβB2P1* overexpression independently increase tumorigenicity, and CRYβB2 overexpression enhances detection of metastatic cells within the liver

**Fig. 4:**
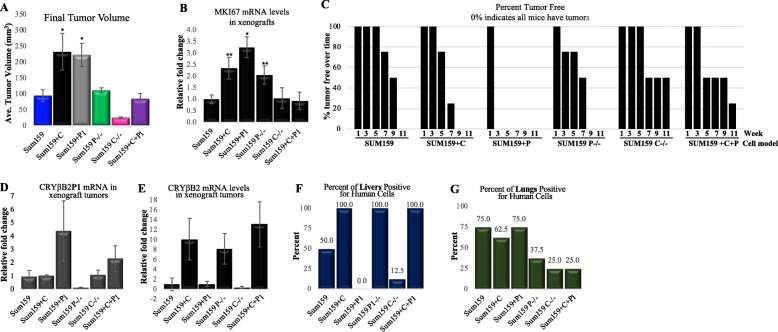
Effect of CRYβB2 and *CRYβB2P1* expression on tumorigenicity. **a** Adult female nude mice were injected into the right abdominal mammary gland with the indicated SUM159 models and orthotopic tumor growth measured over time. **b** qRT-PCR analysis of MKI67 (expression levels within the tumors). **c** Tumor initiation represented as percent of mice tumor free over time. 100% signifies no mice have tumors, 0% indicates all mice have tumors. Data from one representative experiment of three independent experiments, *n* = 5 to 8 mice per treatment group, *p < 0.005, **p < 0.05 compared to SUM159. **d**, **e** qRT-PCR expression levels of the indicated genes in primary xenograft tumor samples. **f**, **g** Detection of human cells within common metastatic sites in xenograft models. Cells were detected via qRT-PCR for the human-specific gene β-2-microglobulin. +C, CRYβB2 overexpression; +P1, *CRYβB2P1* overexpression; P-/-, *CRYβB2P1* knockout; C-/-, CRYβB2 knockout; +C+P1, CRYβB2 and *CRYβB2P1* dual overexpression

Xenografts using the SUM159 models were used to evaluate tumorigenicity and detection of metastatic cells in common sites of metastasis. Similar to proliferation studies, the CRYβB2-overexpressing cells had significantly larger final tumor volume and increased tumor cell proliferation (indicated by increased *Ki67* expression) compared to parental control cells, gene knockouts, and dual gene overexpression models (*p* < 0.005 and *p* < 0.05, *t* test; Fig. 4a, b). *CRYβB2* knockout had the lowest final tumor volume, and no significant change was observed when comparing the control parental cell line which was observed with the *CRYβB2P1* knockout and dual CRYβB2/*CRYβB2P1*-overexpressing cells. Of note, cells overexpressing *CRYβB2P1* alone had significantly larger final tumor volume and significantly increased *Ki67* expression (*p* < 0.005, one-way ANOVA with Bonferroni test). Cells overexpressing *CRYβB2P1* also demonstrated increased tumorigenicity, with tumors readily detected in all animals at 3 weeks post-injection compared to other models that exhibited 100% mice bearing tumors after 8 weeks or later (Fig. 4c). Collectively, these in vivo data suggest additional gene regulatory roles for *CRYβB2P1* that are independent of its ability to regulate CRYβB2.

We confirmed that each cell model retained the desired altered gene expression in the primary tumors via qPCR (Fig. 4e, f), then investigated the presence of metastatic human cells in mouse livers and lungs using human-specific Beta-2-Microglobulin primers. Distinct localization patterns were observed for all models overexpressing CRYβB2; 100% of the cells detected in the liver had either CRYβB2 overexpression or *CRYβB2P1* knockout*,* which significantly increases CRYβB2 expression. Loss of *CRYβB2* or overexpression of *CRYβB2P1* resulted in notably lower levels of detection of cells within the liver (Fig. 4f). This pattern of enhanced detection of cells overexpressing CRYβB2 in the liver was not observed in the lung.

### CRYβB2 overexpression alters breast cancer cell growth behaviors in 3D cell culture

**Fig. 5:**
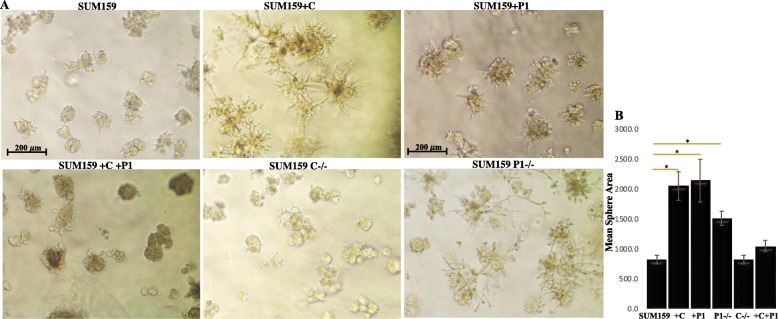
CRYβB2 alters breast cancer cell growth behaviors in 3D cell culture. **a** Cells were grown in Matrigel and live cell growth imaged over time. Representative images were taken at day 10 of growth. Data is one representative assay of a minimum of four independent experiments from both SUM159 and Hs578t models. **b** Data are mean sphere area of the representative 159 models ± SEM. *p < 0.008, one-way ANOVA with Bonferroni test. +C, CRYβB2 overexpression; +P1, *CRYβB2P1* overexpression; P-/-, *CRYβB2P1* knockout C-/-, CRYβB2 knockout; +C+P1, CRYβB2 and *CRYβB2P1* dual overexpression

To directly observe cellular morphology and behaviors, cultures of all the SUM159 and Hs578t cell models were grown in 3D culture and monitored over 10 days via live cell imaging. Consistent with in vivo study results, the size of spheroids was significantly larger in the models overexpressing CRYβB2, *CRYβB2P1*, and *CRYβB2P1* knockout (which increases CRYβB2 levels) compared to the control parental cell lines (*p* < 0.008, one-way ANOVA with Bonferroni test; representative images for the SUM159 models shown in Fig. 5 and Additional file 3: Figure S3 for Hs578t models). The increase in sphere size suggests increased proliferation or survival as observed in xenografts. Indeed, increased expression of *Ki67* was detected via qPCR in *CRYβB2P1-*overexpressing, CRYβB2-overexpressing, and *CRYβB2P1-*knockout models (which increases CRYβB2 expression; *p* < 0.04, *t* test, compared to control parental cell lines, *data not shown*). The most striking observation was the significant increase in invasive structures in cells with high levels of CRYβB2 (CRYβB2 overexpression and *CRYβB2P1* knockouts). Total RNA was extracted from 3D cultures grown for 10 days and analyzed via tumor metastasis and epithelial-mesenchymal transition (EMT) pathway-focused qPCR arrays. Table 2 documents the most significant results, highlighting an overall increase in invasive, EMT, and metastatic genes and a suppression of epithelial and metastatic suppressive genes in cells overexpressing CRYβB2 compared to the control parental SUM159 and Hs578t models.

**Table 2:** Fold change in gene expression of CRYβB2-overexpressing cells compared to control cells

Gene symbol	Name	Fold
IL1RN	Interleukin 1 receptor antagonist	15.07
MMP13	Matrix metallopeptidase 13	12.38
IL1B	Interleukin 1 beta	9.45
TSHR	Thyroid-stimulating hormone receptor	5.92
IGF1	Insulin-like growth factor 1	4.93
CDH11	Cadherin 11, type 2, OB-cadherin	4.66
MMP3	Matrix metallopeptidase 3	4.41
FLT4	Fms-related tyrosine kinase 4	3.87
ITGB3	Integrin, beta 3	3.39
MMP9	Matrix metallopeptidase	3.39
VCAN	Versican	3.14
SOX10	Transcription factor SOX-10	2.61
SSTR2	Somatostatin receptor 2	2.53
CTSL	Cystatin F	2.48
RORB	RAR-related orphan receptor B	2.38
TMEM132A	Glucose-regulated protein, 78 kDa	2.14
CDH6	Cadherin 6, type 2, K-cadherin	− 10.62
FGFBP1	Fibroblast growth factor binding Prot. 1	− 7.78
WNT5B	Wnt family member 5B	− 7.32
CDH1	Cadherin 1, type 1, E-cadherin	− 6.33
COL3A1	Collagen, type III, alpha 1	− 5.63
DSP	Desmoplakin	− 5.09
CDKN2A	Cyclin-dependent kinase inhibitor 2A, p16	− 4.79
MAP1B	Microtubule-associated protein 1B	− 4.22
TGFB2	Transforming growth factor beta 2	− 3.88
MST1R	Macrophage-stimulating 1 receptor	− 3.14
DSC2	Desmocollin 2	− 2.93
WNT11	Wnt family member 11	− 2.75
MTSS1	Metastasis suppressor 1	− 2.68
KISS1R	KiSS-1 metastasis-suppressor receptor	− 2.28
ITGA7	Integrin, alpha 7	− 2.2
FGFR4	Fibroblast growth factor receptor 4	− 2.18

### CRYβB2 overexpression enhances interleukin 6 secretion, signaling, and immune cell attraction

**Fig. 6:**
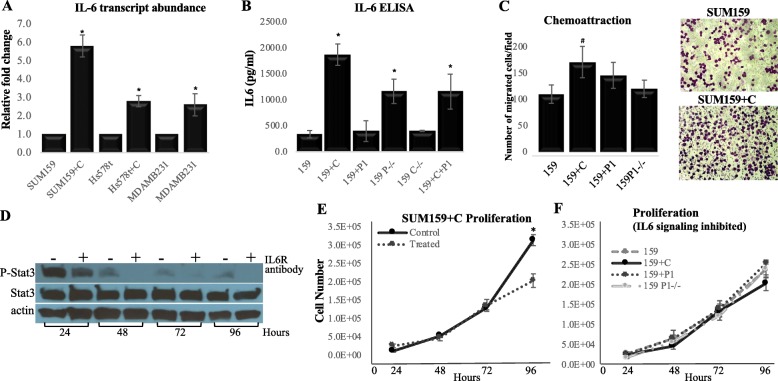
CRYβB2 overexpression significantly increases IL6 secretion and enhances immune cell chemoattraction. **a** qRT-PCR analysis of IL-6 mRNA levels in the indicated cell models +/- CRYβB2 overexpression. **b** IL-6 ELISA. Cells were grown to 80% confluence, washed, then incubated in serum-free media 24 h. Conditioned media collected, concentrated, corrected for total protein then analyzed. Representative model of all three triple-negative breast cancer models. **c** The indicated SUM model was grown to 80% confluence on the bottom of a migration chamber, washed, then incubated in serum-free media 24 h, then U937 cells were plated in serum-free media on the top chamber and allowed to migrate towards the indicated cell model for 4 h. Data are the number of cells migrated per field counted, right panels are representative crystal violet-stained images of migrated cells. **d** Representative immunoblots of SUM159 overexpressing CRYβB2 cells +/- the addition of 75 ng/ml recombinant human IL-6 receptor blocking antibody. **e** Proliferation of SUM159 cells overexpressing CRYβB2 ± 75 ng/ml recombinant human IL-6 receptor blocking antibody, or the indicated cell line treated with the blocking antibody (**f**). Cells were plated at 25,000 cells per well in triplicate and counted every 24 h for 96 h. Blocking antibody was added each day. All data represent mean ± SE of a minimum of three independent experiments. *p < 0.01. ^#^*p =* 0.052. 159, SUM159; +C, CRYβB2 overexpression; +P1, *CRYβB2P1* overexpression; P-/-, *CRYβB2P1* knockout; C-/-, *CRYβB2* knockout; +C+P, CRYβB2 and *CRYβB2P1* dual overexpression

Invasive structures similar to those observed in the CRYβB2-overexpresing models were previously observed using 3D cultures of MCF10AI human breast epithelial cells [38]. These invasive structures were shown to be dependent on IL6 stimulation [38]. IL6 was also previously shown to be one of the most significantly increased inflammatory cytokines in African-American breast cancer patients compared to White patients, and high plasma IL6 levels were identified as a breast cancer risk factor in African-American women [39, 40]. To test if IL6 was contributing to the increased invasive phenotype in CRYβB2-overexpressing cells, IL6 production was tested via qPCR and ELISAs in three triple-negative breast cancer model systems. Overexpression of CRYβB2 significantly induced IL6 expression and secretion in all models tested (*p* < 0.01, *t* test; Fig. 6a, b). Autocrine/paracrine activation of IL6 signaling was confirmed by evaluating STAT3 activation in four triple-negative breast cancer model systems (Additional file 3: Figure S4). Consistent with the role of IL6 in immune cell chemoattraction, in vitro chemoattraction studies show a trend of increased attraction of monocyte-like U937 cells towards cells overexpressing CRYβB2 (Fig. 6c, *p =* 0.052, *t* test).

**Fig. 7:**
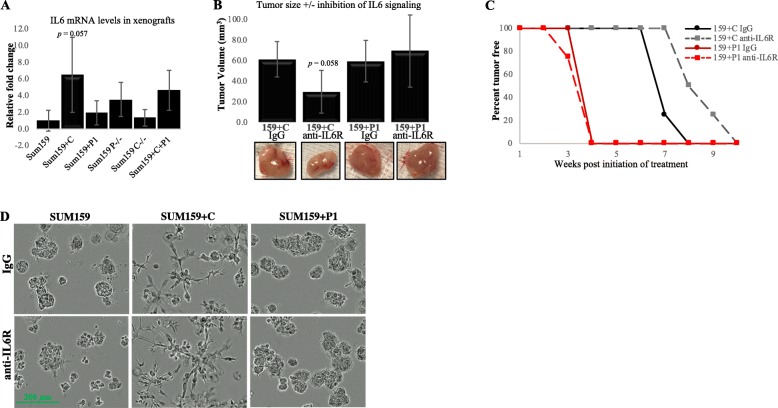
Effect of an IL6 receptor blocking antibody on tumor growth. **a** qPR-PCR analysis of IL6 expression levels in SUM159 xenografts. **b** Female nude mice were injected as described in Fig. 5 using the SUM159+C and SUM159+P cells, which produced the largest tumors. Upon detection of a palpable tumor, an intratumoral injection of an IL6 receptor blocking antibody or non-specific IgG control was injected every day till study end. **c** Tumor initiation over time for each cell line in the presence of the IL6 receptor blocking antibody or control. Data are from one representative experiment (mean ± SD) of two independent experiments, *n* = 8 mice/treatment group for **a** and *n* = 4 mice/treatment group for **b**, **c**. **d** Representative images of cells were grown in Matrigel +/− treatment with an IL6 receptor blocking antibody or non-specific IgG control (added fresh each day) and growth imaged over time. +C, CRYβB2 overexpression; +P1, *CRYβB2P1* overexpression; P-/-, *CRYβB2P1* knockout; C-/-, *CRYβB2* knockout; +C+P, CRYβB2 and *CRYβB2P1* dual overexpression

IL6 also contributes to breast cancer cell proliferation [41]. Correspondingly, inhibition of IL6 signaling using an IL6 receptor blocking antibody demonstrated a decrease in STAT3 activation and the proliferation rate of cells overexpressing CRYβB2 (*p* < 0.01, *t* test; Fig. 6d, e). Inhibition of IL6 signaling in the CRYβB2-overexpressing cells resulted in no significant difference in growth rates between all CRYβB2/*CRYβB2P1* modified models tested (Fig. 6f). Enhanced IL6 production was correspondingly detected in xenograft tumors with increased levels of CRYβB2 (Fig. 7a). Xenograft studies were repeated using the IL6R blocking antibody in vivo, and results demonstrate inhibition of IL6 signaling reduced tumorigenesis of SUM159 cells overexpressing CRYβB2, but had no significant effect on final tumor volume in *CRYβB2P1-*overexpressing cells (Fig. 7b, c). While tumor proliferation was reduced in xenografts, the invasive phenotype induced by CRYβB2 overexpression was not decreased when IL6 was inhibited in 3D cultures (Fig. 7d).

## Discussion

This study demonstrates that both CRYβB2 and *CRYβB2P1* promote tumor growth, but their mechanisms for tumor promotion are likely distinct. Overexpression of CRYBB2, but not *CRYβB2P1*, induces IL6 secretion, cell proliferation in 2D cultures, an invasive phenotype in 3D cultures, and a consistent homing of metastatic cells to the liver. Conversely, *CRYβB2P1* overexpression promotes tumorigenicity via increasing proliferation, while suppressing *CRYβB2* expression. The suppression of *CRYβB2 by CRYβB2P1* may be critical for cell function, as our results demonstrate overexpression of both CRYβB2 and *CRYβB2P1* suppresses cell proliferation and tumor growth. This supports the idea that *CRYβB2P1* may function as an antisense regulator to the parental gene *CRYβB2.*

Previous functional studies have suggested similar results where a gene and its pseudogene have common mechanisms but mutual inhibition. For example, Korneev et al. showed that translation of the neural nitric oxide synthase (NOS) protein was inhibited by expression of the NOS pseudogene [42]*.* We hypothesize that the suppression of CRYβB2 expression by the pseudogene may have evolved during gene replication as a protective mechanism to inhibit inappropriate cellular proliferation, but this mechanism has been manipulated by the cancer cell to ensure proliferation and tumor progression. As previously stated, simultaneous overexpression of both *CRYβB2* and *CRYβB2P1* is inhibitory to cell proliferation and tumorigenesis, suggesting a mechanistically distinct, but functional redundancy between the ancestral/parental gene and pseudogene. It is also clear that the mechanism by which CRYβB2 and *CRYβB2P1* affect tumor promotion vary independently, given the lack of direct correlation/relationship between the expression levels of *CRYβB2* and *CRYβB2P1* in TCGA breast cancer samples. Other studies such as Duret et al. have found evidence that pseudogenes can evolve independently from their parental genes and have independent functions, which may be relevant to the *CRYβB2* and *CRYβB2P1* relationship [43].

Another major conclusion that can be drawn from our investigation is the distinction of the race-related expression of *CRYβB2* and *CRYβB2P1.* First, our data highlight that expression of the pseudogene, *CRYβB2P1,* is associated with Black/African-American breast cancer patients compared to White and Asian patient samples, and *CRYβB2P1*expression levels are higher compared to *CRYβB2* in all TCGA breast cancer samples. Other studies have highlighted *CRYβB2* as a health disparity gene in breast cancer, but our data suggest technical difficulties in distinguishing between the expression levels of the two genes and that the more likely disparity target is *CRYβB2P1* [7, 11, 12, 14–17]. Second, our data suggest *CRYβB2P1* functions as a ncRNA in triple-negative breast cancers to alter transcription.

Pseudogenes are copies of protein-coding genes that no longer produce the same functional product as their parental gene, but still share a high sequence similarity, and can thus regulate or mediate the function of their parental genes through mechanisms such as the generation of ncRNA. Pseudogenes can be transcribed in parallel with their parental genes, or with their own tissue or temporal specific patterns [28]. Evidence shows that pseudogenes perform vital roles in regulating normal tissue growth and the development of some diseases, especially cancers [28]. They can serve as antisense regulatory transcripts or miRNA decoy, produce siRNAs or ncRNAs, and encode short proteins [44]. For example, Lethe is a pseudogene that produces a ncRNA. The pseudogene ncRNA is selectively induced by pro-inflammatory cytokines via NFκB and functions in negative feedback signaling to NFκB [45]. The genomic-wide effect of *CRYβB2P1* is currently under investigation in our laboratory. Of note, a review of seven cancer lines in the publicly available University of California, Santa Cruz, genome browser database indicates *CRYβB2P1* is active (marked with active histone mark, H3K4me3) and rich in transcription factor and chromatin regulatory marks, while not many regulatory marks are present for *CRYβB2*. These data strongly suggest *CRYβB2P1* ncRNA may act in cis at the *CRYβB2* locus and/or function in trans genome-wide.

One limitation of the current study is the focus on triple-negative breast cancers. We have previously reported *CRYβB2* as one of four genes significantly associated with African-American race and survival in luminal A breast cancers [7]. Data presented herein clearly show a role for CRYβB2 independent of *CRYβB2P1* in the promotion of breast cancer, including increased proliferation, tumorigenesis, and invasive behaviors. Whether *CRYβB2P1* alters *CRYβB2* expression, or has tumor-promoting effects independent of *CRYβB2*, specifically in luminal cells was not investigated. Another constraint of our study is that although triple-negative breast cancer is a heterogeneous disease, we restricted our TCGA and cell line analyses to basal-like subtypes [46–49]. While multiple subtype classification methods for TNBCs currently exist, the basal subtype comprises over 70% of the TNBC subtype. Restricting our analyses to basal-like subtypes reduces experimental heterogeneity and increases generalizability of our results. In addition to cancer subtype specificity, we acknowledge the potential that exists that the influence of CRYβB2 and *CRYβB2P1* on tumor cell behaviors is tissue dependent. Pilot studies in our laboratory show *CRYβB2* but not *CRYβB2P1* is expressed significantly higher in pancreatic cancer cell lines compared to primary pancreatic cells (Additional file 3: Figure S5).

The data presented herein demonstrate a set of biological functions and physiological consequences of high CRYβB2 protein expression in breast cancer models. Overexpression of CRYβB2 increased IL6 production, upregulated the expression of proliferative genes, and increased proliferation of breast cancer cells in vitro and in vivo*.* CRYβB2 also induced EMT/metastatic phenotypes in triple-negative breast cancer cells including the upregulation of a set of genes known to increase tumor metastasis in vivo. Sox10 was one gene of note that was significantly upregulated in CRYβB2-overexpressing cells. A recent eloquent study by Dravis et al. demonstrated that in both mouse and human tumors, SOX10 expression correlates with stem/progenitor identity, dedifferentiation, and invasive characteristics, and DNA binding motifs for SOX transcription factors are enriched in stem/progenitor cells [50]. This data suggests the potential for the upregulation of SOX10 and IL6 in CRYβB2-overexpressing cells mediating the trans-differentiation to an invasive, EMT-like phenotype.

## Conclusions

In summary, our findings support a mechanistic role in racial differences for *CRYβB2*, but suggest that *CRYβB2P1* is a relevant disparities target. We provide novel data emphasizing the need to distinguish the biological effects of CRYβB2 and those of the ncRNA, *CRYβB2P1*, as overexpression of either gene enhances tumor progression*.* Our studies demonstrate that *CRYβB2P1* can enhance tumorigenesis in vivo, and loss of *CRYβB2P1* expression results in significantly increased CRYβB2 levels. To our knowledge, we are the first to report physiological consequences of breast cancer cells that have high CRYβB2 expression including increased tumor proliferation, IL6 secretion, enhanced metastatic homing to the liver, increased expression of metastatic and EMT-associated genes, and invasive cellular behaviors. These data are highly relevant as they demonstrate novel molecular mechanism of two understudied molecules for potential therapeutic development. Targeting CRYβB2 and *CRYβB2P1* may assist in reducing the disparate survival outcomes observed between Black and White American breast cancer patients, or may better identify those patients most at risk for more aggressive disease.

## Additional files

Additional file 1: Table S1. Breast Cancer Cell lines and their associated race of origin and subtype. (PDF 82 kb)

Additional file 2: Table S2. qPCR Primer Sequences (PDF 37 kb)

Additional file 3: Figure S1. Distribution of each gene among subtype and race using linear regression: conditioning on race (a) or subtype (b). *CRYβB2* *adjusted *p* = 0.00427; *CRYβB2P1* *adjusted *p*=7.7E-05, **adjusted *p* =1.3E-02. e *CRYβB2P1* *adjusted *p* = 0.0043, **adjusted *p* = 0.0008. Distribution of *CRYβB2* and *CRYβB2P1* among race within subtype (c) and *CRYβB2P1* among age/menopausal status (d). Significant results for *CRYβB2* *adjusted *p* = 0.0304, and for *CRYβB2P1* *adjusted *p* = 2.4E-06, ^#^adjusted *p* = 5.3E-03, and **adjusted *p* = 2.3E-04. d *CRYβB2P1* distribution among age before conditioning for race: *adjusted *p* = 0.0334, **adjusted *p* = 0.0231, ^#^adjusted *p* = 0.0052, ^##^adjusted *p* = 0.0269, and (e) subtype after conditioning for race: * adjusted *p* = 0.0071, ** adjusted *p* = 0.0369, and ^#^adjusted *p* = 0.0257. Young <40 yrs, Pre = pre-menopausal 40-46, Peri = peri-menopausal 46-55, and Post = post-menopausal >55 yrs. Figure S2. Distribution of transcript localization for each gene following subcellular fractionalization of RNAs. RNA was isolated and separated into cytosolic and nuclear subcellular fractions from proliferating cells. U6 and ACTB expression show successful separation of the nuclear and cytosolic subcellular compartments, respectively. 159 = SUM159, P-/- = *CRYβB2P1* knockout, C-/- = *CRYβB2* knockout, cyto = cytosolic fraction, nuc = nuclear fraction. Figure S3. CRYβB2 alters breast cancer cell growth behaviors in 3D cell culture. a Cells were grown in Matrigel and imaged on day 8. Data is one representative assay of a minimum of four independent experiments from Hs578t models. Figure S4. Cells were grown to 80% confluence, washed, then incubated in serum-free media 24 h. Images are representative immunoblots from the indicated models of control parental or CRYβB2-overexoressing cells. All data represent a minimum of three independent experiments. +C = CRYβB2 overexpression. Figure S5. *CRYβB2* and *CRYβB2P1* expression patterns of pancreatic cancer cell models. qRT-PCR analysis of the indicated cell lines. HPNE = hTERT-HPNE non-cancerous pancreatic ducal cells. Remaining cell models are pancreatic cancer cell lines. (PDF 5920 kb)

## Data Availability

Data used in this study are included in this published article and its supplementary files.

## Abbreviations

ANOVA: Analysis of variance
CRISPR: Clustered regularly interspaced short palindromic repeats
CRYβB2: Crystallin beta B2
*CRYβB2P*: Crystallin beta B2 pseudogene 1
EMT: Epithelial-mesenchymal transition
HSD: Honestly significant difference
IL6: Interleukin 6
ncRNA: Non-coding RNA
NFκB: Nuclear factor kappa-light-chain-enhancer of activated B cells
SOX10: SRY-Box 10
STAT3: Signal transducer and activator of transcription 3
TCGA: The Cancer Genome Atlas
