# Supplementary material for: A functional role for the cancer disparity-linked genes, CRYβB2 and CRYβB2P1, in the promotion of breast cancer
Source: Breast Cancer Res. 2019 Sep 11;21:105. doi: 10.1186/s13058-019-1191-3 (PMC6739962; doi:10.1186/s13058-019-1191-3)
Supplement: Supplementary file 1 — Table S1. Breast Cancer Cell lines and their associated race of origin and subtype. (PDF 82 kb) [file 13058_2019_1191_MOESM1_ESM.pdf]

Table S1. Breast Cancer Cell Lines

Cell Line	Molecular Subtype	Race
MCF7	Luminal A	white
T47D	luminal A	white
HCC1500	Luminal A	black
HCC1428	Luminal A	white
SUM185	Luminal A	white
ZR75.1	Luminal B	white
BT474	Luminal B	white
MDAMB361	Luminal B	white
HCC1143	Basal, Triple Negative A	white
HCC1806	Basal, Triple Negative A	black
MCF10AII	Basal, Triple Negative A	white
BT-20	Basal, Triple Negative A	white
HCC1937	Basal, Triple Negative A	white
MDAMB468	Basal, Triple Negative A	black
HCC70	Basal, Triple Negative A	black
HCC2157	Basal, Triple Negative A	black
SUM102	Basal, Triple Negative B	white
SUM149	Basal, Triple Negative B	white
SUM159	Basal, Triple Negative B	white
SUM1315mo	Basal, Triple Negative B	white
MDAMB231	Basal, Triple Negative B	white
Hs578t	Basal, Triple Negative B	white
MDAMB157	Basal, Triple Negative B	black
BT549	Basal, Triple Negative B	white

Table S2. Primer sequences

Gene	Entrez Gene ID	Sequence (5´- 3´)
CRYBB2	1415	AGAAGGCAGGTTCTGTCCTA<br>GGTACTCACCCTTCTCAAACAC
CRYBB2P1	1416	AGAAAACAGCTCACGTCTATGG<br>TAGCTCACCCAGCGTGTA
RPL13a	23521	CCAAGATGCACTATCGGAAGAA<br>CTTGAGGACCTCTGTGAACTTG
PP1a	5419	TGGCAAGACCAGCAAGAA<br>CTCCTGAGCTACAGAAGGAATG
ACTB	60	TGCTGGTTGCTGCTTACA<br>GCCTATCTCCTGTCGCATTATAG
B2M	567	GCCGTGTGAACCATGTGACTTT<br>CCAAATGCGGCATCTTCAAA
MKI67	4288	TGACCCTGATGAGAAAGCTCAA<br>CCCTGAGCAACACTGTCTTTT
IL6	3569	GGAGACTTGCCTGGTGAAA<br>CTGGCTTGTTCCTCACTACTC
