# Supplementary material for: A functional role for the cancer disparity-linked genes, CRYβB2 and CRYβB2P1, in the promotion of breast cancer
Source: Breast Cancer Res. 2019 Sep 11;21:105. doi: 10.1186/s13058-019-1191-3 (PMC6739962; doi:10.1186/s13058-019-1191-3)
Supplement: Supplementary file 2 — Table S2. qPCR Primer Sequences (PDF 37 kb) [file 13058_2019_1191_MOESM2_ESM.pdf]

Table S2. Primer sequences

Gene	Entrez Gene ID	Sequence (5´- 3´)
CRYBB2	1415	AGAAGGCAGGTTCTGTCCTA<br>GGTACTCACCCTTCTCAAACAC
CRYBB2P1	1416	AGAAAACAGCTCACGTCTATGG<br>TAGCTCACCCAGCGTGTA
RPL13a	23521	CCAAGATGCACTATCGGAAGAA<br>CTTGAGGACCTCTGTGAACTTG
PP1a	5419	TGGCAAGACCAGCAAGAA<br>CTCCTGAGCTACAGAAGGAATG
ACTB	60	TGCTGGTTGCTGCTTACA<br>GCCTATCTCCTGTCGCATTATAG
B2M	567	GCCGTGTGAACCATGTGACTTT<br>CCAAATGCGGCATCTTCAAA
MKI67	4288	TGACCCTGATGAGAAAGCTCAA<br>CCCTGAGCAACACTGTCTTTT
IL6	3569	GGAGACTTGCCTGGTGAAA<br>CTGGCTTGTTCCTCACTACTC
