# Supplementary material for: A functional role for the cancer disparity-linked genes, CRYβB2 and CRYβB2P1, in the promotion of breast cancer
Source: Breast Cancer Res. 2019 Sep 11;21:105. doi: 10.1186/s13058-019-1191-3 (PMC6739962; doi:10.1186/s13058-019-1191-3)
Supplement: Supplementary file 3 — Figure S1. Distribution of each gene among subtype and race using linear regression: conditioning on race (a) or subtype (b). CRYβB2 *adjusted p = 0.00427; CRYβB2P1 *adjusted p=7.7E-05, **adjusted p =1.3E-02. e CRYβB2P1 *adjusted p = 0.0043, **adjusted p = 0.0008. Distribution of CRYβB2 and CRYβB2P1 among race within subtype (c) and CRYβB2P1 among age/menopausal status (d). Significant results for CRYβB2 *adjusted p = 0.0304, and for CRYβB2P1 *adjusted p = 2.4E-06, #adjusted p = 5.3E-03, and **adjusted p = 2.3E-04. d CRYβB2P1 distribution among age before conditioning for race: *adjusted p = 0.0334, **adjusted p = 0.0231, #adjusted p = 0.0052, ##adjusted p = 0.0269, and (e) subtype after conditioning for race: * adjusted p = 0.0071, ** adjusted p = 0.0369, and #adjusted p = 0.0257. Young <40 yrs, Pre = pre-menopausal 40-46, Peri = peri-menopausal 46-55, and Post = post-menopausal >55 yrs. Figure S2. Distribution of transcript localization for each gene following subcellular fractionalization of RNAs. RNA was isolated and separated into cytosolic and nuclear subcellular fractions from proliferating cells. U6 and ACTB expression show successful separation of the nuclear and cytosolic subcellular compartments, respectively. 159 = SUM159, P-/- = CRYβB2P1 knockout, C-/- = CRYβB2 knockout, cyto = cytosolic fraction, nuc = nuclear fraction. Figure S3. CRYβB2 alters breast cancer cell growth behaviors in 3D cell culture. a Cells were grown in Matrigel and imaged on day 8. Data is one representative assay of a minimum of four independent experiments from Hs578t models. Figure S4. Cells were grown to 80% confluence, washed, then incubated in serum-free media 24 h. Images are representative immunoblots from the indicated models of control parental or CRYβB2-overexoressing cells. All data represent a minimum of three independent experiments. +C = CRYβB2 overexpression. Figure S5. CRYβB2 and CRYβB2P1 expression patterns of pancreatic cancer cell models. qRT-PCR analysis of the i [file 13058_2019_1191_MOESM3_ESM.pdf]

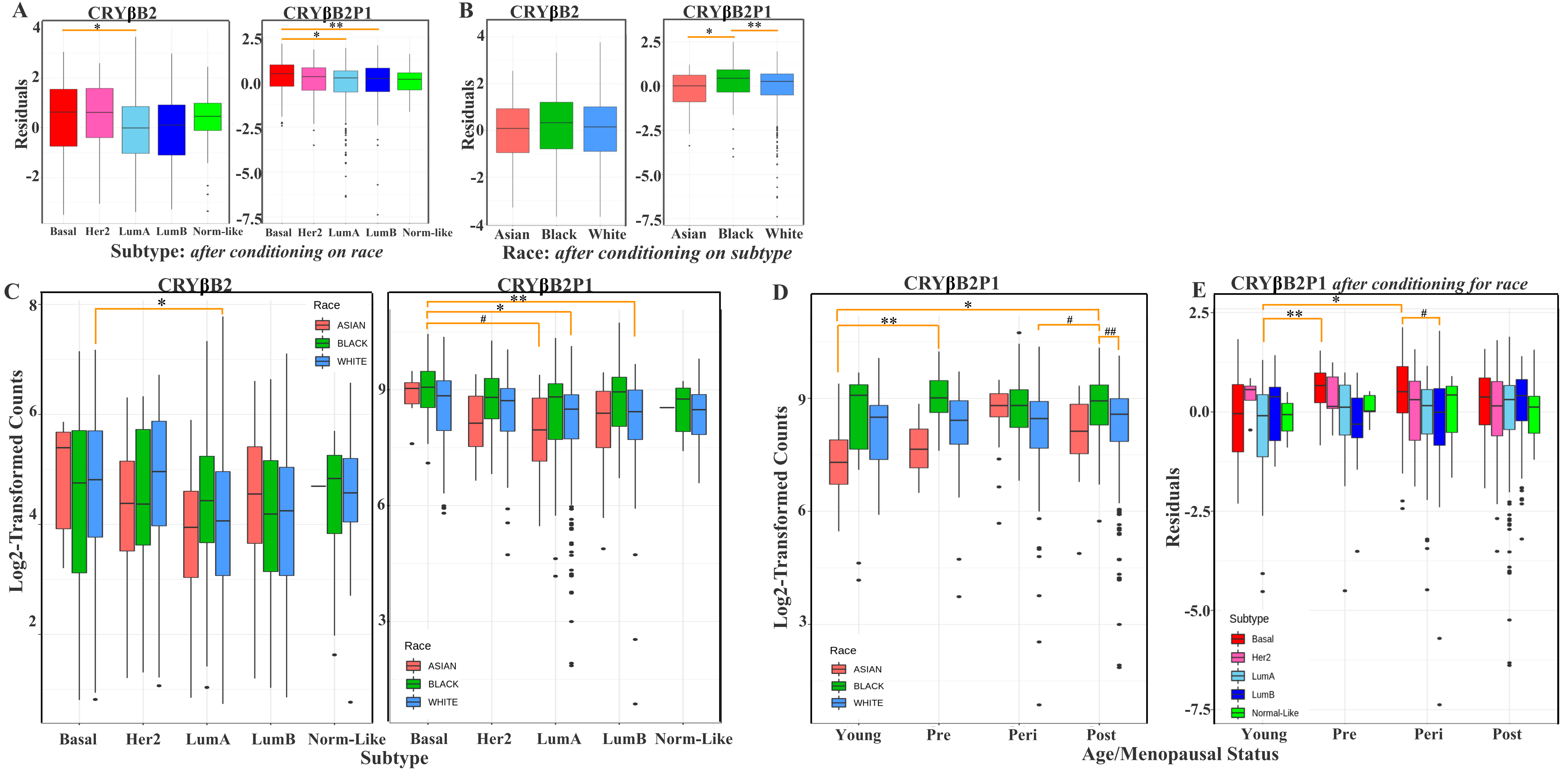


**Figure S1.** Distribution of each gene among subtype and race using linear regression: conditioning on race (a) or subtype (b). *CRYβB2* *adjusted $p$ = 0.00427; *CRYβB2P1* *adjusted $p$=7.7E-05, **adjusted $p$ =1.3E-02. e *CRYβB2P1* *adjusted $p$ = 0.0043, **adjusted $p$ = 0.0008. Distribution of *CRYβB2* and *CRYβB2P1* among race within subtype (c) and *CRYβB2P1* among age/menopausal status (d). Significant results for *CRYβB2* *adjusted $p$ = 0.0304, and for *CRYβB2P1* *adjusted $p$ = 2.4E-06, #adjusted $p$ = 5.3E-03, and **adjusted $p$ = 2.3E-04. d *CRYβB2P1* distribution among age before conditioning for race: *adjusted $p$ = 0.0334, **adjusted $p$ = 0.0231, #adjusted $p$ = 0.0052, ##adjusted $p$ = 0.0269, and (e) subtype after conditioning for race: * adjusted $p$ = 0.0071, ** adjusted $p$ = 0.0369, and #adjusted $p$ = 0.0257. Young <40 yrs, Pre = pre-menopausal 40-46, Peri = peri-menopausal 46-55, and Post = post-menopausal >55 yrs.

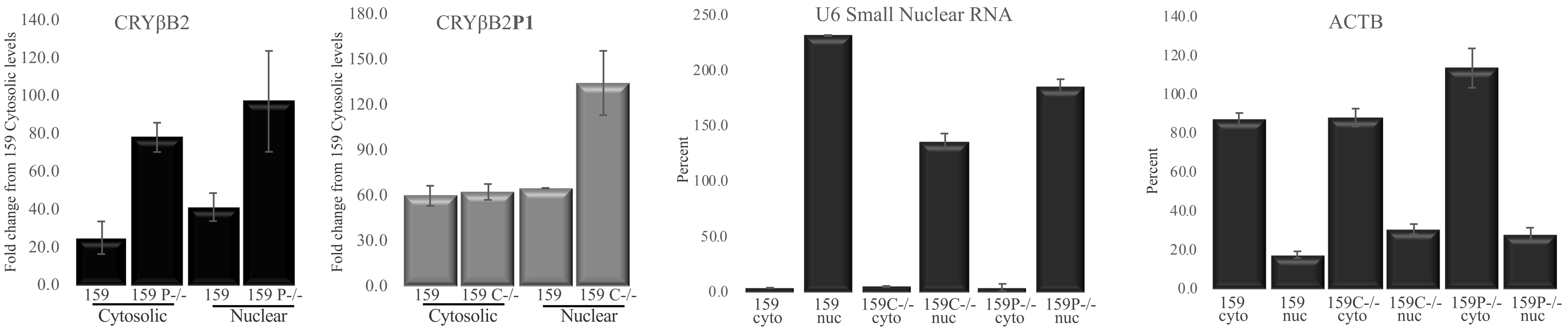


**Figure S2.** Distribution of transcript localization for each gene following subcellular fractionalization of RNAs. RNA was isolated and separated into cytosolic and nuclear subcellular fractions from proliferating cells. U6 and ACTB expression show successful separation of the nuclear and cytosolic subcellular compartments, respectively. 159 = SUM159, P-/- = *CRYβB2P1* knockout, C-/- = *CRYβB2* knockout, cyto = cytosolic fraction, nuc = nuclear fraction.

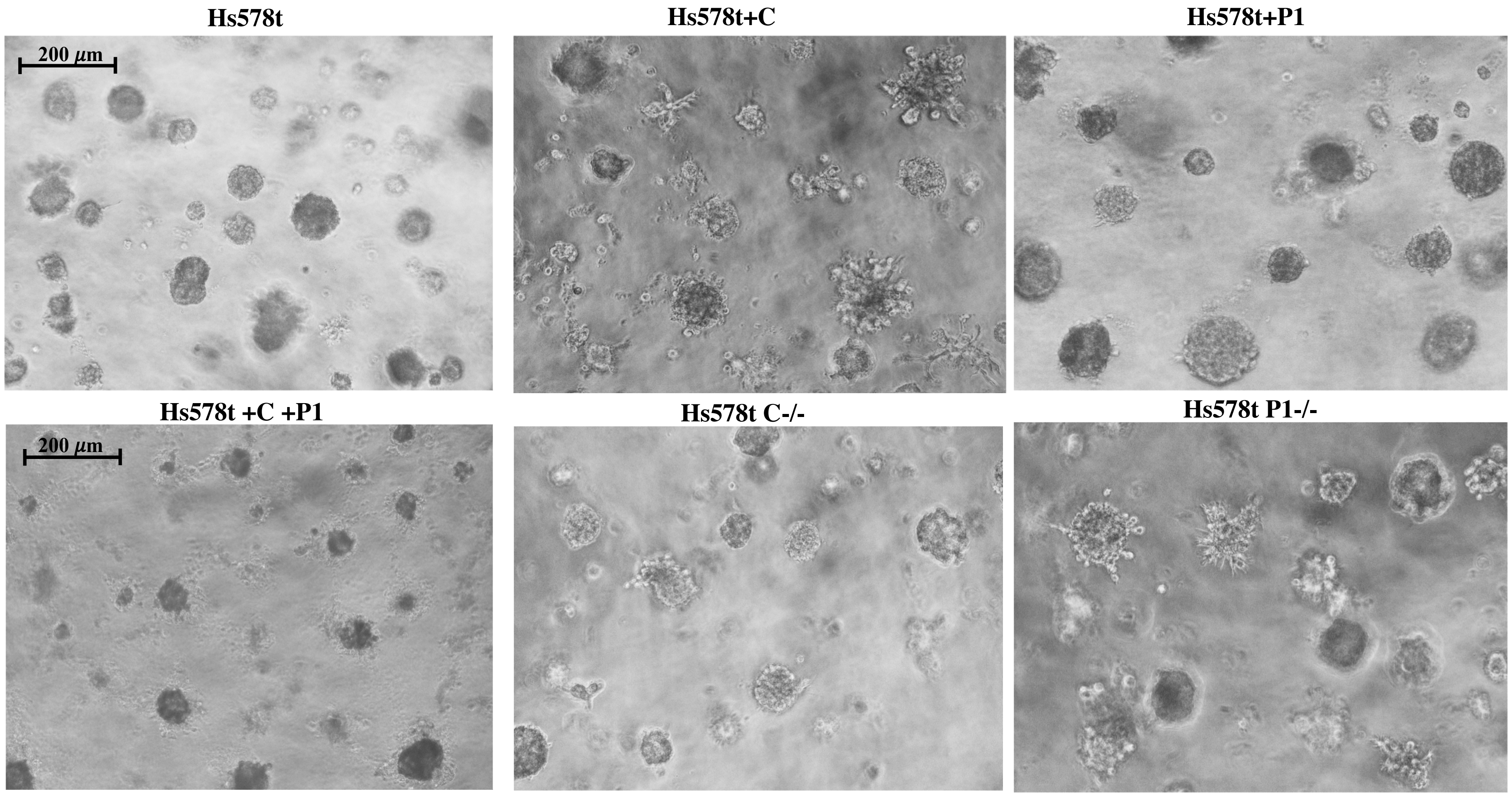


**Fig. S3. CRYβB2 alters breast cancer cell growth behaviors in 3D cell culture.** Cells were grown in Matrigel and imaged over time. Representative images were taken at day ten of growth. Data is one representative assay of a minimum of four independent experiments from Hs578t models. +C = CRYβB2 overexpression, +P1 = *CRYβB2P1* overexpression, P-/- = *CRYβB2P1* knockout C-/- = CRYβB2 knockout, +C+P1 = CRYβB2 and *CRYβB2P1* dual overexpression

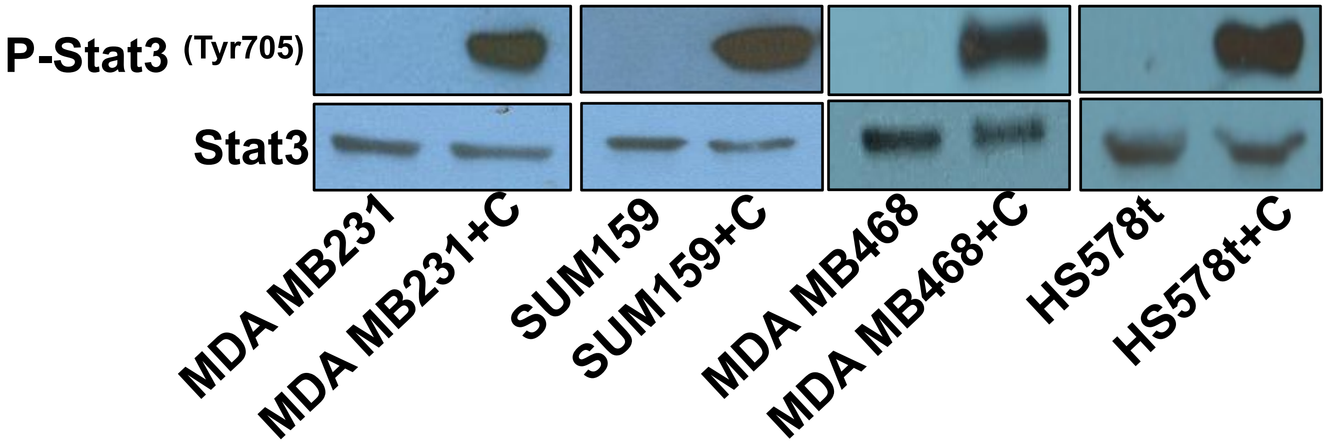


**Figure S4.** Cells were grown to 80% confluence, washed, then incubated in serum-free media 24 h. Images are representative immunoblots from the indicated models of control parental or CRYβB2-overexoressing cells. All data represent a minimum of three independent experiments. +C = CRYβB2 overexpression.

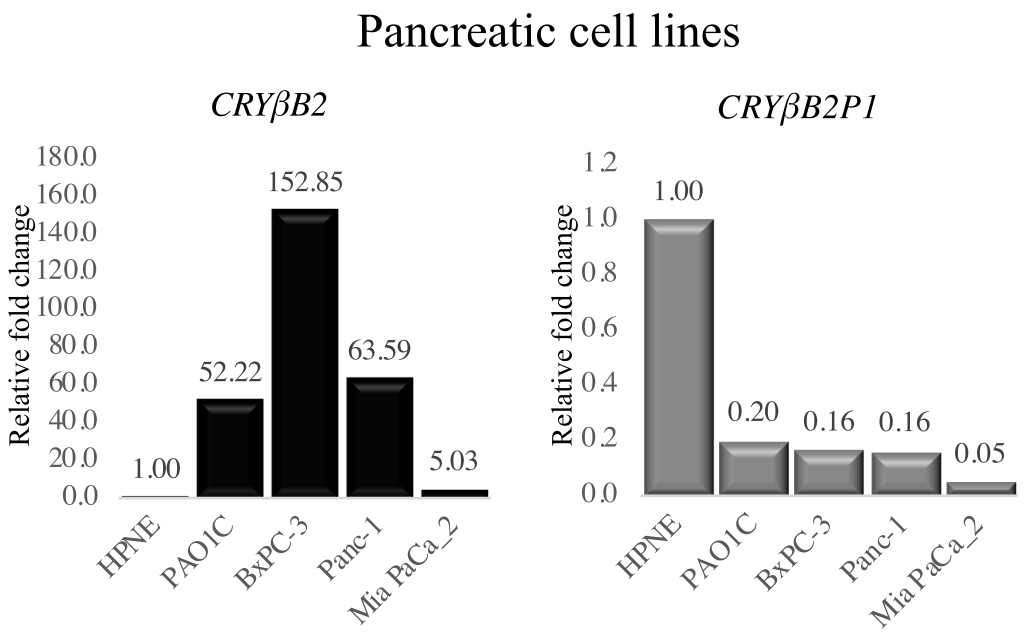


**Figure S5.** *CRYβB2* and *CRYβB2P1* expression patterns of pancreatic cancer cell models. qRT-PCR analysis of the indicated cell lines. HPNE = hTERT-HPNE non-cancerous pancreatic ducal cells. Remaining cell models are pancreatic cancer cell lines.
